# Multi-Omics Insights into the Metabolic Basis of Variation in 4-Alkyl Branched-Chain Fatty Acids in Meat from Chinese Sheep Breeds

**DOI:** 10.3390/foods15213964

**Published:** 2026-11-04

**Authors:** Jinlong Li, Bin Chen, Wujun Liu

**Affiliations:** College of Animal Science, Xinjiang Agricultural University, Urumqi 830052, China; lijinlong0120@126.com (J.L.); chenbin_1998@163.com (B.C.)

**Keywords:** 4-methyloctanoic acid, 4-ethyloctanoic acid, 4-methylnonanoic acid, branched-chain amino acids, propanoate metabolism, integrated multi-omics analysis

## Abstract

4-Alkyl branched-chain fatty acids (BCFAs), including 4-methyloctanoic acid (4-MOA), 4-ethyloctanoic acid (4-EOA), and 4-methylnonanoic acid (4-MNA), are important contributors to the characteristic odor of sheep meat. Their concentrations and composition are closely related to meat flavor quality and consumer acceptance. Understanding the mechanisms that regulate BCFA formation and accumulation is therefore important for improving sheep meat flavor. In this study, the concentrations of these three BCFAs were measured in muscle samples from Altay (ALT), Turpan (TLF), Inner Mongolian (NM), Tan, and Hu sheep. Untargeted muscle metabolomic data from ALT and TLF sheep were integrated with publicly available datasets, including metabolomic data from Tan sheep under different feeding conditions and transcriptomic and metabolomic data from Hu and Zang sheep. These datasets were used to investigate BCFA-related metabolic networks and candidate regulatory factors associated with ecological environment, nutritional conditions, and genetic background. The results show clear differences in BCFA accumulation among the sheep populations. All three BCFAs were below the limits of detection in ALT, TLF and NM sheep. Both 4-MOA and 4-EOA were detected in Tan sheep, whereas all three BCFAs were detected in Hu sheep. The concentrations of 4-MOA and 4-EOA in Hu sheep were approximately 14.4- and 12.5-fold higher than those in Tan sheep, respectively (*p* < 0.001). Multi-omics integration showed that BCFA-related metabolic variation was mainly associated with branched-chain amino acid metabolism, short-chain carbon metabolism, the tricarboxylic acid (TCA) cycle, and lipid metabolism. *MMUT*, *ACAT1*, and *EHHADH* were identified as candidate regulatory genes. These genes are involved in methylmalonyl-CoA conversion, acetyl-CoA/acetoacetyl-CoA metabolism, and fatty acid β-oxidation, respectively. Changes in energy demand, substrate utilization, and precursor availability may alter the partitioning of BCFA precursor carbon between central energy metabolism and lipid metabolism, thereby affecting BCFA formation and accumulation. In summary, *MMUT*, *ACAT1*, and *EHHADH* may influence the formation and accumulation of 4-MOA, 4-EOA, and 4-MNA by regulating key metabolic reactions and carbon partitioning between the TCA cycle and lipid metabolism. Ecological environment and dietary conditions may further contribute to this process by modifying energy metabolism and precursor substrate supply. These findings provide a theoretical basis for understanding BCFA-related flavor formation and for improving sheep meat flavor through genetic selection and nutritional regulation.

## 1. Introduction

Sheep meat flavor is an important factor affecting eating quality and consumer acceptance. Compared with beef and pork, sheep meat has a distinct species-specific flavor that is shaped by several biochemical processes, including lipid oxidation, amino acid degradation, the Maillard reaction, and the conversion of endogenous flavor precursors in muscle tissues [1,2,3]. Among the compounds involved, 4-alkyl branched-chain fatty acids (BCFAs) are important chemical contributors to the characteristic odor of sheep meat. The major BCFAs include 4-methyloctanoic acid (4-MOA), 4-ethyloctanoic acid (4-EOA), and 4-methylnonanoic acid (4-MNA) [4]. Previous studies using gas chromatography–olfactometry (GC-O), odor activity values (OAVs), and aroma recombination and omission experiments have confirmed that several BCFAs make important contributions to the characteristic “muttony” odor of sheep meat [2,3,4]. Therefore, understanding the formation, conversion, and accumulation of BCFAs is important for clarifying the biochemical basis of characteristic sheep meat odor.

The formation and accumulation of BCFAs in sheep meat are influenced by multiple factors, including genetic background, ecological environment, and nutritional conditions. Previous studies have shown that differences in meat quality and flavor among Tibetan sheep breeds are closely associated with amino acid degradation, carbohydrate metabolism, and lipid metabolism [5]. Clear differences have also been observed in the metabolite profiles of sheep meat from different geographical origins, suggesting that ecological origin can influence flavor-related metabolic processes [6]. In addition, dietary composition and feeding systems may affect the production and accumulation of flavor precursors by altering rumen microbial composition, volatile fatty acid supply, and host lipid metabolism [7,8]. Studies in Australian sheep have associated fat BCFA concentrations with age and pre-slaughter nutrition [9]. A New Zealand feeding trial also reported lower concentrations of characteristic short-chain BCFAs in pasture-fed lambs than in lambs receiving lucerne- or maize-based concentrate diets [10]. Thus, variation in BCFA formation under different genetic, environmental, and nutritional conditions may reflect the combined effects of multiple metabolic processes, but the underlying regulatory mechanisms have not yet been systematically characterized.

BCFA formation involves several interconnected metabolic processes, including branched-chain amino acid (BCAA) degradation, the utilization of rumen fermentation products, fatty acid synthesis, and energy metabolism. In ruminants, dietary nutrients are fermented by rumen microorganisms to produce volatile fatty acids such as propionate. After entering host tissues, propionate can be converted to propionyl-CoA and further enter methylmalonyl-CoA-related metabolic pathways [4,11]. Methylmalonyl-CoA is then converted to succinyl-CoA by methylmalonyl-CoA mutase (MMUT), allowing it to enter the tricarboxylic acid (TCA) cycle and participate in energy metabolism. This process may also influence the distribution of branched-chain carbon substrates among different metabolic pathways. In addition, the degradation of BCAAs such as valine and isoleucine generates branched-chain carbon skeletons that can enter propionate-related metabolic networks, thereby linking amino acid metabolism with BCFA formation [12,13]. BCFA accumulation may therefore depend on the dynamic partitioning of precursor carbon among energy oxidation, lipid synthesis, and branched-chain fatty acid formation.

Although these metabolic processes provide a theoretical basis for understanding BCFA formation, the metabolic basis of differences in BCFA accumulation among sheep from different sources remains poorly understood. Most previous studies have focused on a single breed, a specific feeding condition, or individual metabolic processes related to sheep meat flavor. A comprehensive understanding is still lacking of how precursor availability, carbon flow, and related metabolic networks jointly regulate BCFA accumulation under different genetic backgrounds, ecological environments, and nutritional conditions [14,15]. The development of multi-omics approaches provides a useful strategy for investigating the biological basis of such complex traits. By integrating metabolomic and transcriptomic information, changes in metabolites, metabolic pathways, and candidate genes can be evaluated together, providing a more complete view of the potential biological mechanisms underlying flavor-related traits [16,17].

Therefore, this study focuses on Chinese indigenous sheep with different genetic backgrounds and ecological origins. Quantitative analysis of odor-related BCFAs was combined with muscle metabolomics and publicly available multi-omics datasets to investigate the potential metabolic basis of differences in BCFA accumulation associated with genetic background, ecological environment, and nutritional conditions. The study aimed to identify key metabolic processes and candidate regulatory factors involved in BCFA formation, thereby providing a theoretical basis for understanding the formation of characteristic sheep meat flavor and for improving meat quality through breed selection and nutritional regulation.

## 2. Materials and Methods

### 2.1. Study Design and Data Sources

This study combined quantification of odor-related 4-alkyl branched-chain fatty acids (BCFAs), untargeted muscle metabolomics, and reanalysis of independent public omics datasets. BCFA quantification involved five sheep populations: Altay (ALT), Turpan (TLF), Inner Mongolian (NM), Tan, and Hu sheep. Untargeted muscle metabolomic analysis was further performed on samples from ALT and TLF sheep. The public datasets included muscle transcriptomic and metabolomic data from Hu and Zang sheep published by Wang et al. [18], and muscle metabolomic data from Tan sheep under different feeding systems published by Yang et al. [19]. Zang sheep were included only in the public-data reanalysis and were not involved in our animal sampling or BCFA quantification. The public Hu and Tan datasets originated from experiments independent of our sampling cohort. These datasets were used to explore candidate metabolic processes potentially associated with BCFA formation and accumulation.

All procedures involving animals and sample collection were approved by the Animal Welfare and Ethics Committee of Xinjiang Agricultural University (Approval No. 2023053) and were conducted in accordance with ISO/TS 34700:2016 [20]. Ten healthy male sheep from each of the ALT, TLF, NM, Tan, and Hu populations were selected. The animals were 7–8 months old and had a mean body weight of 42.48 ± 4.57 kg. ALT and TLF sheep are adapted to the cold and hot climates of their respective regions of origin. Table 1 summarizes the breed names, animal sources, rearing and sampling locations, sampling seasons, feeding systems, and main dietary components of the five populations to clarify their genetic, environmental, and nutritional backgrounds.

Longissimus dorsi (LD) muscle samples were collected following the same commercial slaughter procedure and stored at −80 °C until analysis. The concentrations of 4-MOA, 4-EOA, and 4-MNA were quantified in LD samples from ALT, TLF, NM, Tan, and Hu sheep. Untargeted metabolomic analysis was further performed on LD samples from ALT and TLF sheep.

NM sheep sampled in Sunite Left Banner, Inner Mongolia, were included to broaden the geographical coverage of the BCFA comparison beyond the Xinjiang ALT and TLF populations. Untargeted metabolomic analysis focused on LD samples from ALT and TLF sheep with contrasting ecological adaptation backgrounds.

To investigate metabolic characteristics related to BCFA formation, the multi-omics dataset of longissimus dorsi muscle from Hu and Zang sheep published by Wang et al. [18] was reanalyzed. Differentially expressed genes, differential metabolites, and KEGG pathway annotations were integrated for association analysis.

To evaluate the association between dietary nutritional conditions and BCFA-related metabolic networks, the LD metabolomic dataset of Tan sheep raised under different feeding systems published by Yang et al. [19] was reanalyzed.

### 2.2. Quantification of Mutton Odor-Related 4-Alkyl Branched-Chain Fatty Acids

The concentrations of 4-MOA, 4-EOA, and 4-MNA in sheep LD muscle were determined according to the method described by Wang et al. [21]. Boron trifluoride–methanol solution, undecanoic acid (C11:0), and diethyl ether were purchased from Shanghai Aladdin Biochemical Technology Co., Ltd. (Shanghai, China). Briefly, 0.5 g of freeze-dried longissimus dorsi muscle was mixed with 2 mL of 2.0 mol/L NaOH–methanol solution (Molekula Ltd., Newton Aycliffe, UK) and hydrolyzed at 110 °C for 2 h. After cooling to room temperature, 2 mL of 14% BF3–methanol solution was added, and the mixture was incubated at 80 °C for 15 min to obtain the corresponding fatty acid methyl ester derivatives. Undecanoic acid (C11:0) was added as an internal standard. The derivatives were then extracted twice with diethyl ether, and the organic phases were combined and concentrated to approximately 1 mL under a stream of nitrogen.

The derivatives were analyzed using an Agilent 7890B gas chromatograph coupled to a 5977B mass selective detector (Agilent Technologies, Santa Clara, CA, USA). Chromatographic separation was performed on a DB-WAX capillary column (30 m × 0.25 mm, 0.5 μm film thickness; Agilent Technologies, Santa Clara, CA, USA). The injection volume was 1 μL, with a split ratio of 10:1, and the inlet temperature was maintained at 250 °C. The oven temperature was initially set at 75 °C and held for 2 min, then increased to 230 °C at a rate of 10 °C/min and held for 7 min. High-purity helium was used as the carrier gas at a flow rate of 0.65 mL/min. Mass spectrometry was performed using an electron ionization (EI) source at an electron energy of 70 eV, and the transfer line temperature was set at 250 °C. The LODs used for 4-MOA, 4-EOA, and 4-MNA were 0.15, 0.11, and 0.11 μg/g dry weight, respectively. The corresponding LOQs were set at three times the LODs, giving values of 0.45, 0.33, and 0.33 μg/g dry weight, respectively. Results below the analyte-specific LOD were reported as not detected (ND). The RSDs of the QC samples were below 10%, and C11:0 internal-standard recoveries ranged from 90% to 118%.

### 2.3. Metabolomic Analysis of Longissimus Dorsi Muscle from Altay and Turpan Sheep

Ten LD samples from each of the ALT and TLF groups were selected for untargeted metabolomic analysis, which was performed by Wuhan Metware Biotechnology Co., Ltd. (Wuhan, China). Methanol, acetonitrile, and formic acid were purchased from Shanghai Aladdin Biochemical Technology Co., Ltd. (Shanghai, China). Before analysis, the samples were thawed on ice and ground under liquid nitrogen. Approximately 20 mg of tissue was weighed and extracted with 400 μL of 70% methanol aqueous solution containing internal standards. The samples were shaken at 1500 r/min for 5 min and then incubated on ice for 15 min. After centrifugation at 12,000 r/min for 10 min at 4 °C, 300 μL of the supernatant was collected and kept at −20 °C for 30 min. The samples were then centrifuged again, and 200 μL of the supernatant was used for LC–MS analysis.

Metabolomic analysis was performed using a Vanquish ultra-high-performance liquid chromatography system coupled to a Q Exactive HF-X high-resolution mass spectrometer (Thermo Scientific, Waltham, MA, USA). Chromatographic separation was carried out on a Waters ACQUITY Premier HSS T3 column (1.8 μm, 2.1 × 100 mm; Waters Corporation, Milford, MA, USA). Mobile phase A consisted of water containing 0.1% formic acid, whereas mobile phase B consisted of acetonitrile containing 0.1% formic acid. The column temperature was maintained at 40 °C, the flow rate was 0.4 mL/min, and the injection volume was 4 μL. The gradient program was as follows: 5%–20% B from 0 to 2 min, 20%–60% B from 2 to 5 min, 60%–99% B from 5 to 6 min, and 99% B from 6 to 7.5 min. The mobile phase was then returned to 5% B and equilibrated until 10 min. Mass spectrometric data were acquired in both positive and negative electrospray ionization (ESI) modes over an *m*/*z* range of 75–1000. Metabolites were identified using TraceFinder 3.2 (Thermo Fisher Scientific, Waltham, MA, USA). Quality control (QC) samples were prepared from all samples and used to evaluate the stability of the analytical process. Raw data processing and metabolite annotation were performed by the service provider using standard pipelines.

### 2.4. Acquisition and Reanalysis of Public Multi-Omics Data from Hu and Zang Sheep

The muscle transcriptomic and metabolomic data from Hu and Zang sheep were obtained from the publicly available study published by Wang et al. [18]. In the original study, RNA sequencing and untargeted metabolomics were performed on muscle samples from the two groups. The RNA-seq data were deposited in the NCBI Sequence Read Archive under BioProject PRJNA1081808. Because Hu and Zang sheep have different genetic backgrounds, this multi-omics dataset was used to compare gene expression and metabolic characteristics between the two sheep populations and to provide additional evidence for the analysis of BCFA-related metabolic networks. For the transcriptomic component, published differential-expression results and gene annotations from Wang et al. [18] were used for pathway mapping and integration with metabolomic data. The reported fold changes and statistical results were retained without recalculating differential expression. Raw-read processing, alignment, and expression quantification were not repeated in this study. The published metabolite abundance data and annotations were used for metabolomic reanalysis. The transcriptomic and metabolomic data were derived from the same set of biological samples, and the metabolomic dataset included six samples each from Hu and Zang sheep. Metabolite abundance data were normalized before principal component analysis (PCA) to compare the overall metabolic profiles of the two groups. Differential metabolites were identified based on the differential analysis results and were further examined by hierarchical clustering and KEGG pathway annotation for subsequent integrative analysis.

### 2.5. Integrated Transcriptomic and Metabolomic Analysis of Hu and Zang Sheep

Integrated analysis was performed using the transcriptomic and metabolomic data from Hu and Zang sheep. Differentially expressed genes, differential metabolites, and KEGG pathway annotations were analyzed together. The distributions of differential genes and metabolites across KEGG pathways were compared to identify representative pathways supported by both transcriptomic and metabolomic evidence. Pathways related to amino acid metabolism, short-chain organic acid metabolism, lipid metabolism, and energy metabolism were prioritized and visualized using bubble plots.

Representative differential genes, differential metabolites, and related metabolic nodes were further integrated based on KEGG pathway annotations and known biochemical reactions to construct a candidate metabolic network. Known biochemical reactions, associations supported by omics data, and candidate links inferred from established metabolic relationships were distinguished in the network. Metabolic intermediates that were not directly detected were included only to indicate pathway connections and were not used for abundance analysis or between-group comparisons.

### 2.6. Reanalysis of Public Targeted Metabolomic Data from Tan Sheep

The Tan sheep metabolomic data were obtained from the publicly available study published by Yang et al. [19]. In this study, hydrophilic metabolite data from Supplementary Table S3 were used, including eight muscle samples from the concentrate-fed (CF) group and eight from the pasture-fed (PF) group. The KEGG and HMDB annotations provided in the original study were retained for subsequent analysis.

Metabolite abundance data were transformed using log_2_(x + 1) and then normalized. Principal component analysis (PCA) was performed to compare the overall metabolic profiles of the CF and PF groups. Fold changes and log_2_FC values were calculated using PF/CF, and the *p* values obtained from between-group comparisons were adjusted using the Benjamini–Hochberg method. Representative metabolites were selected from pathways related to BCAA degradation, propionate metabolism, and the TCA cycle. These metabolites were further compared with related metabolic nodes identified in other datasets to evaluate changes in candidate metabolic modules under different nutritional conditions.

### 2.7. Statistical Analysis

Statistical analyses were performed in R (version 4.5.3; R Foundation for Statistical Computing, Vienna, Austria). Two-group comparisons were conducted using two-sided Welch’s *t*-tests. For multiple testing, *p*-values were adjusted using the Benjamini–Hochberg method to control the false discovery rate (FDR). The adjusted *p*-values are reported as FDR values. Results are presented as the mean ± standard deviation (SD). For omics analyses involving multiple comparisons, statistical significance was defined as FDR < 0.05. Findings with nominal *p* < 0.05 but FDR ≥ 0.05 were interpreted as exploratory observations. Metabolite abundances were scaled to unit variance before PCA, and hierarchical clustering heatmaps were generated using standardized abundances. Differentially abundant metabolites were selected using the criteria of variable importance in projection (VIP) > 1, *p* < 0.05, and FC > 1.2 or FC < 0.8333. The ALT/TLF, Hu/Zang, and Tan CF/PF datasets were analyzed independently, with statistical comparisons made between groups from the same study. Cross-study comparisons used metabolite annotations, pathway assignments, and the direction of change observed within each dataset. Sample abundance matrices were kept separate, and signal intensities were not compared across studies. The proposed network combined these findings with known biochemical relationships.

## 3. Results

### 3.1. Differences in Mutton Odor-Related Branched-Chain Fatty Acids Among Sheep Breeds

**Figure 1:**
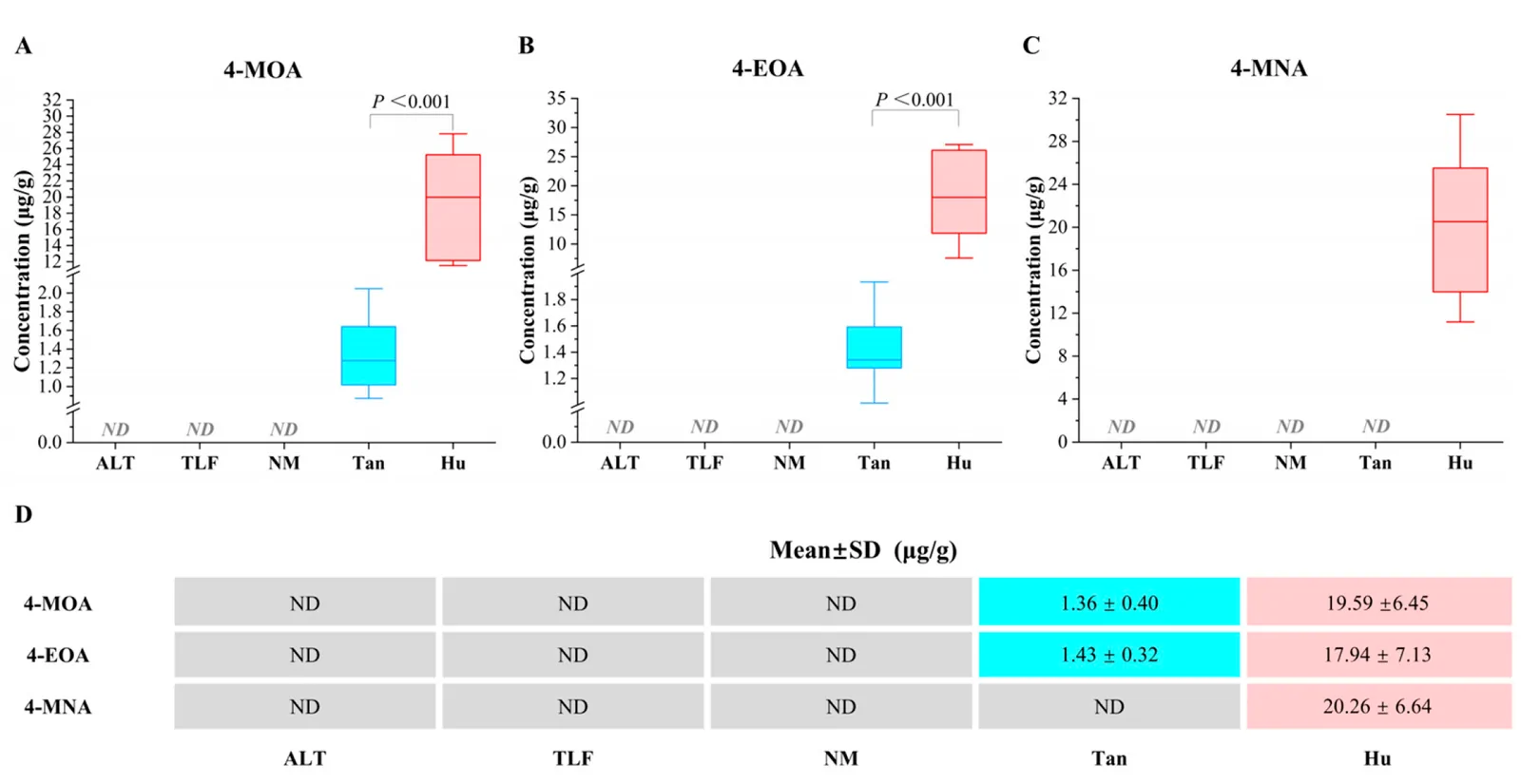
Differences in mutton odor-associated branched-chain fatty acids among ALT, TLF, NM, Tan, and Hu sheep. (**A**–**C**) Concentrations of 4-MOA, 4-EOA, and 4-MNA, respectively, in LD. (**D**) Summary of the concentrations of the three compounds across the five sheep breeds, expressed as the mean ± SD (μg/g). ND, not detected; concentrations were below the detection limit of the analytical method.

Quantitative analysis of 4-MOA, 4-EOA, and 4-MNA in the LD muscle of ALT, TLF, NM, Tan, and Hu sheep showed clear differences among the groups. None of the three BCFAs were detected in ALT, TLF, or NM sheep. In Tan sheep, 4-MOA and 4-EOA were detected, whereas 4-MNA was not detected. All three BCFAs were detected in Hu sheep (Figure 1A–C). The concentrations of 4-MOA and 4-EOA in Hu sheep were approximately 14.4- and 12.5-fold higher than those in Tan sheep, respectively (*p* < 0.001) (Figure 1D).

### 3.2. Differences in Longissimus Dorsi Metabolomic Profiles Between Altay and Turpan Sheep

**Figure 2:**
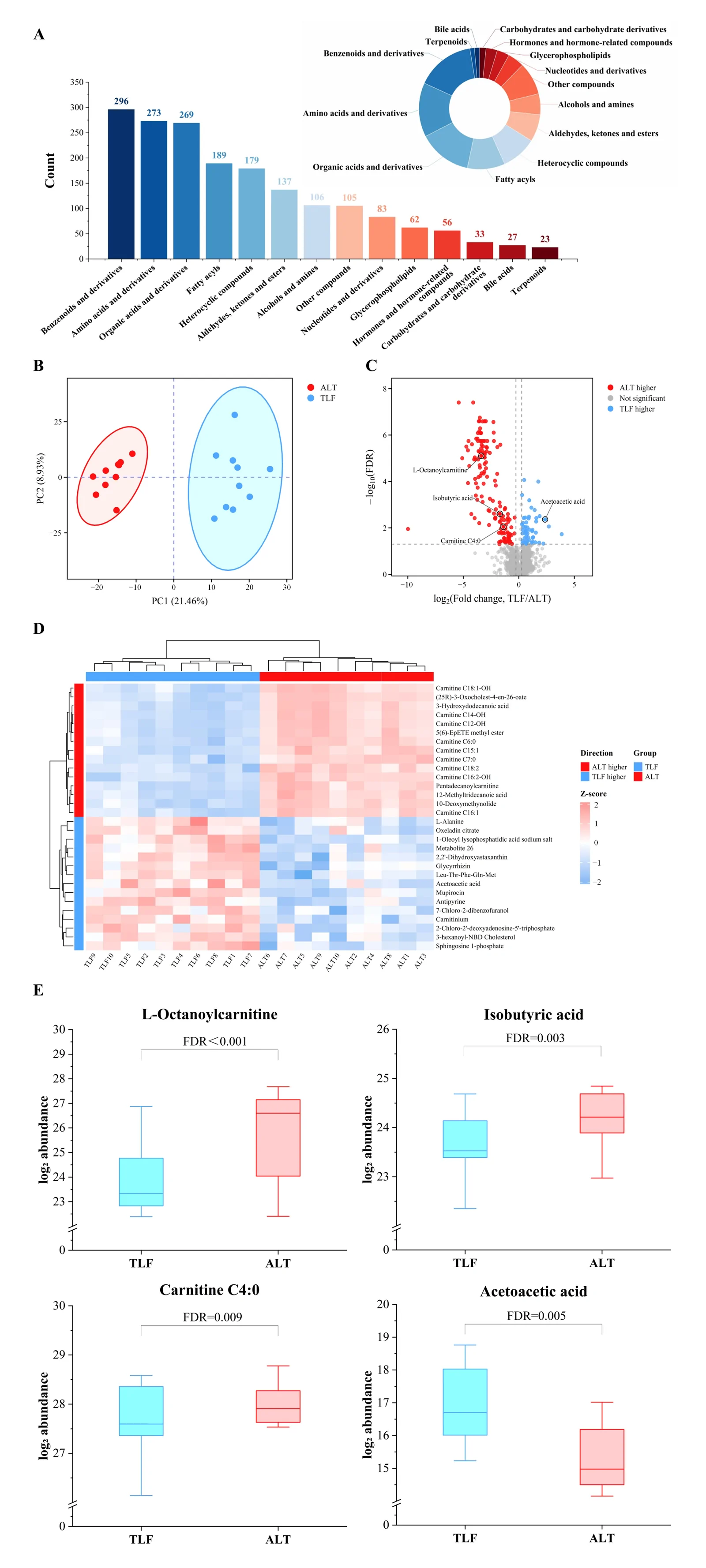
Metabolic profile differences and differential metabolites in the longissimus dorsi muscle of ALT and TLF sheep. (**A**) Chemical classification of the detected metabolites according to metabolite superclass, with the bar chart showing the number of metabolites assigned to each category and the inset doughnut chart showing their relative composition. (**B**) PCA of the muscle metabolomic profiles of ALT and TLF sheep. (**C**) Volcano plot of differential metabolites between ALT and TLF sheep. (**D**) Hierarchical clustering heatmap of 30 representative differential metabolites. (**E**) Boxplots showing the abundance distributions of L-octanoylcarnitine, isobutyric acid, carnitine C4:0, and acetoacetic acid in ALT and TLF sheep. Dashed lines in (B) indicate zero scores for PC1 and PC2. In (C), the vertical and horizontal dashed lines indicate the fold-change and FDR cutoffs, respectively.

Untargeted metabolomic analysis of longissimus dorsi muscle from ALT and TLF sheep was performed using UPLC–MS/MS. A total of 1749 metabolites were detected, including 296 benzene and substituted derivatives, 273 amino acids and derivatives, 269 organic acids and derivatives, 189 fatty acyls, and 179 heterocyclic compounds (Figure 2A). Principal component analysis (PCA) showed a clear tendency of separation between ALT and TLF samples in the principal component space. PC1 and PC2 explained 21.46% and 8.93% of the total variance, respectively, and samples within each group showed relatively tight clustering (Figure 2B).

Volcano plot analysis identified 202 differential metabolites between the ALT and TLF groups (Figure 2C). Among them, L-octanoylcarnitine, isobutyric acid, and carnitine C4:0 showed higher abundances in the ALT group, whereas acetoacetic acid was more abundant in the TLF group. Hierarchical clustering of representative differential metabolites also showed a relatively clear separation between ALT and TLF samples (Figure 2D). Further analysis of four representative metabolites showed that L-octanoylcarnitine (FDR < 0.001), isobutyric acid (FDR = 0.003), and carnitine C4:0 (FDR = 0.009) were significantly increased in the ALT group, whereas acetoacetic acid was significantly increased in the TLF group (FDR = 0.005) (Figure 2E).

KEGG pathway annotation of the differential metabolites showed that the metabolic differences between ALT and TLF sheep were mainly associated with lipid metabolism, amino acid metabolism, and other metabolic processes (Supplementary Figure S1).

### 3.3. Metabolomic Differences in the Longissimus Dorsi Muscle of Tan Sheep Under Different Feeding Systems

**Figure 3:**
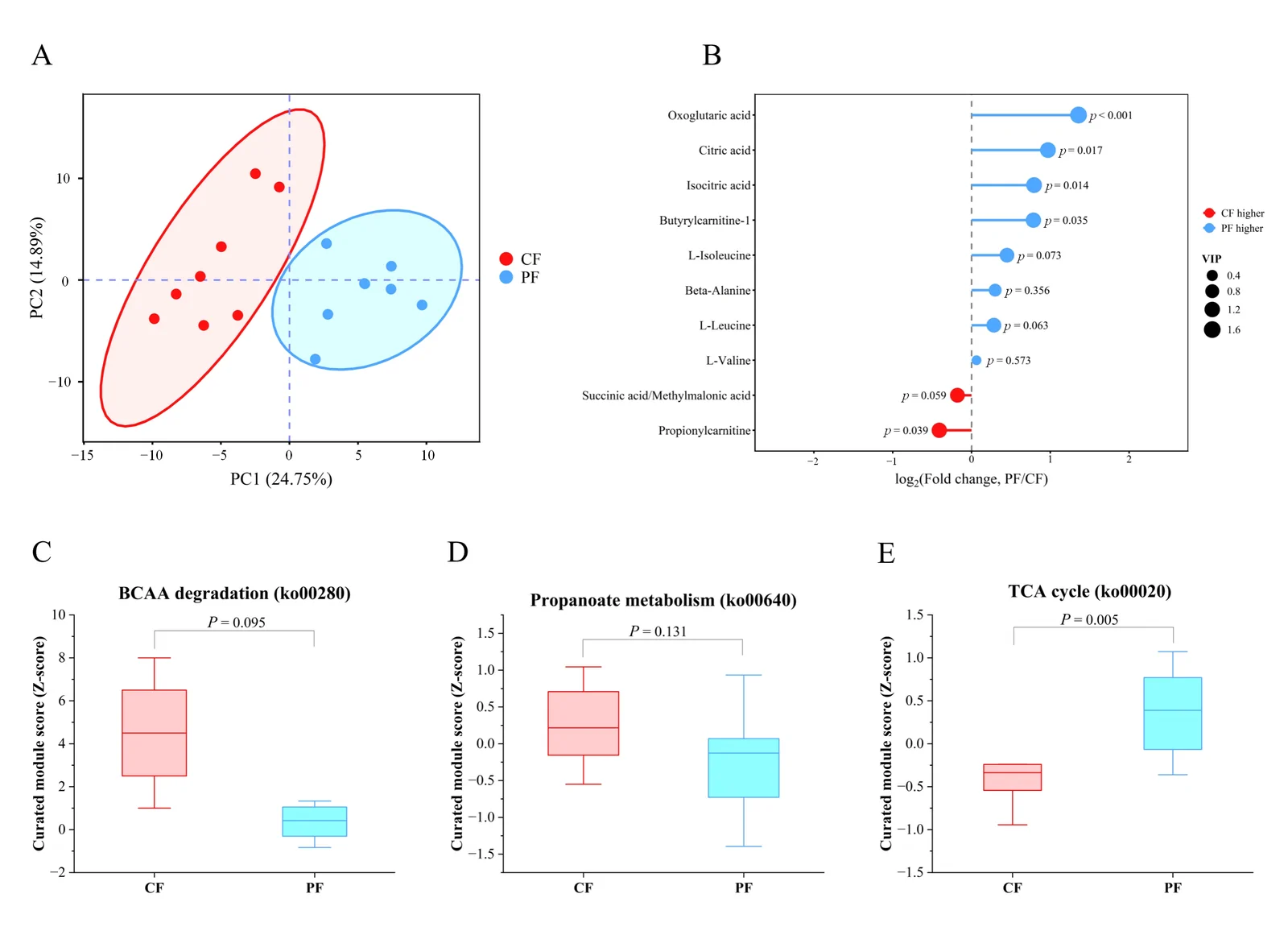
Metabolic alterations in the longissimus dorsi muscle of Tan sheep under different feeding conditions. (**A**) PCA of muscle metabolomic profiles from CF and PF Tan sheep. (**B**) Effect-size plot of representative metabolites associated with the proposed metabolic model. (**C**–**E**) Effect-size plot of representative metabolites associated with BCFA-related metabolic pathways. Dashed lines in (A) indicate zero scores on the PC1 and PC2 axes. The vertical dashed line in (B) indicates log_2_C = 0 (FC = 1), representing no fold change between the two groups.

Principal component analysis (PCA) of the longissimus dorsi (LD) metabolome showed a clear separation between the concentrate-fed (CF) and pasture-fed (PF) Tan sheep groups. PC1 and PC2 explained 24.75% and 14.89% of the total variance, respectively (Figure 3A).

Among the representative differential metabolites, propionylcarnitine showed a higher abundance in the CF group, whereas citric acid, isocitric acid, and butyrylcarnitine were more abundant in the PF group (Figure 3B). L-valine, L-isoleucine, L-leucine, beta-alanine, and succinic acid/methylmalonic acid also showed varying degrees of differences between the two groups.

Composite scores were calculated using selected representative metabolites involved in BCAA degradation, propionate metabolism, and the TCA cycle (Figure 3C). No significant differences were observed in the composite scores for BCAA degradation or propionate metabolism between the CF and PF groups, whereas the TCA cycle score differed significantly between the two groups (*p* = 0.005).

### 3.4. Metabolomic Differences in the Longissimus Dorsi Muscle Between Hu and Zang Sheep

**Figure 4:**
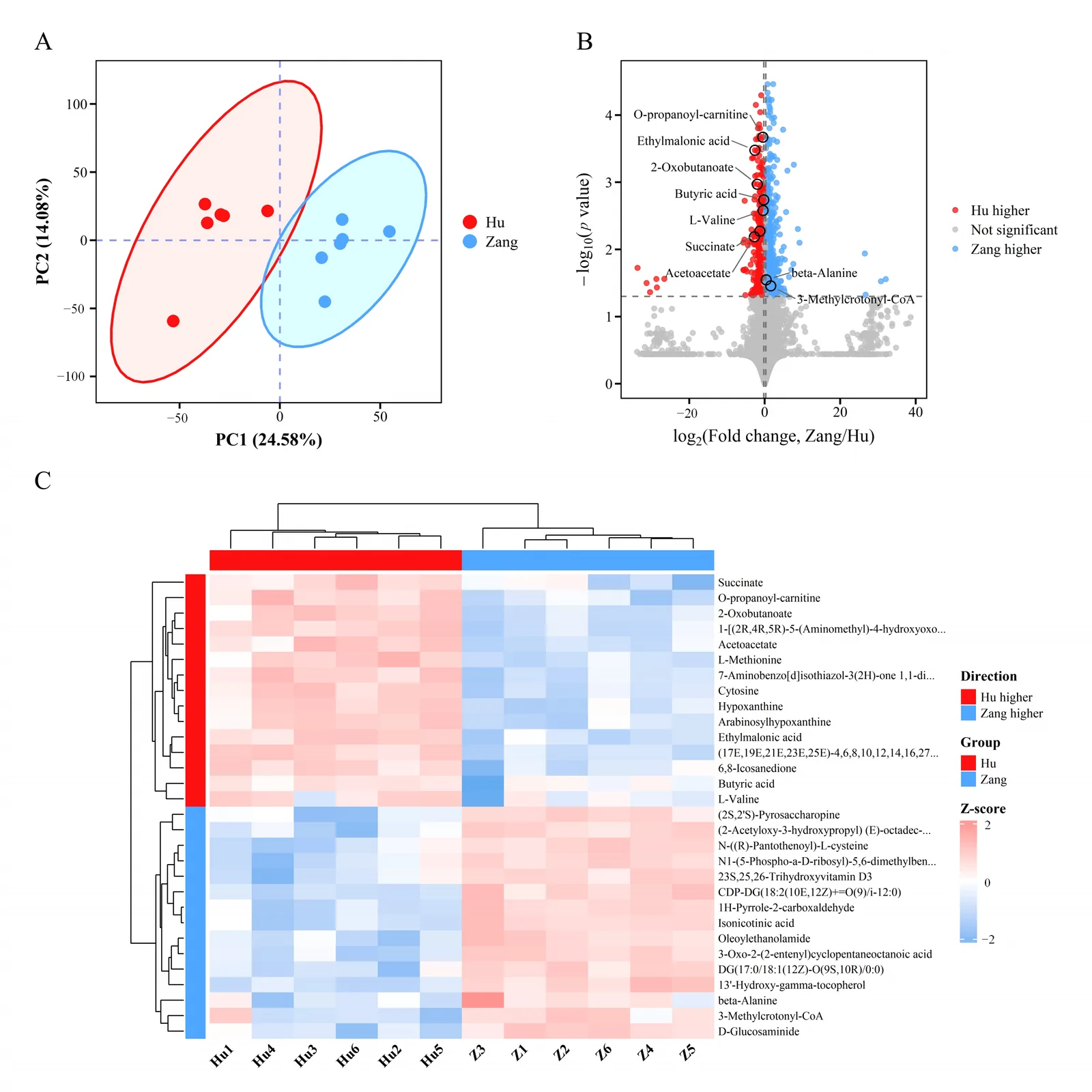
Metabolic profile differences and representative differential metabolites in the longissimus dorsi muscle of Hu and Zang sheep. (**A**) PCA of the muscle metabolomic profiles of Hu and Zang sheep, showing the overall distribution of the two groups. (**B**) Volcano plot of differential metabolites between Hu and Zang sheep. (**C**) Hierarchical clustering heatmap of 30 representative differential metabolites. Dashed lines in (A) indicate zero scores for PC1 and PC2. In (B), the vertical dashed lines indicate the fold-change cutoffs, and the horizontal dashed line indicates the p-value cutoff.

Publicly available metabolomic data were used to compare the metabolic characteristics of longissimus dorsi muscle between Hu and Zang sheep. Principal component analysis (PCA) showed a tendency of separation between the two groups in the principal component space (Figure 4A).

Volcano plot analysis identified 790 differential metabolites, of which 321 showed higher abundance in the Hu group and 469 showed higher abundance in the Zang group (Figure 4B). O-propanoyl-carnitine, ethylmalonic acid, 2-oxobutanoate, butyric acid, succinate, acetoacetate, and L-valine were more abundant in the Hu group, whereas beta-alanine and 3-methylcrotonyl-CoA were more abundant in the Zang group.

Hierarchical clustering showed distinct metabolite abundance patterns between Hu and Zang sheep (Figure 4C). Several representative differential metabolites were related to branched-chain amino acid degradation, propionate metabolism, and the TCA cycle.

KEGG pathway annotation showed that the differential metabolites were mainly associated with amino acid degradation, organic acid metabolism, short-chain carbon metabolism, and lipid-related metabolic processes. The major pathways included valine, leucine and isoleucine degradation, propionate metabolism, butyrate metabolism, fatty acid metabolism, and the TCA cycle (Supplementary Figure S2).

### 3.5. Integrated Transcriptomic and Metabolomic Analysis of the LD in Hu and Zang Sheep

**Figure 5:**
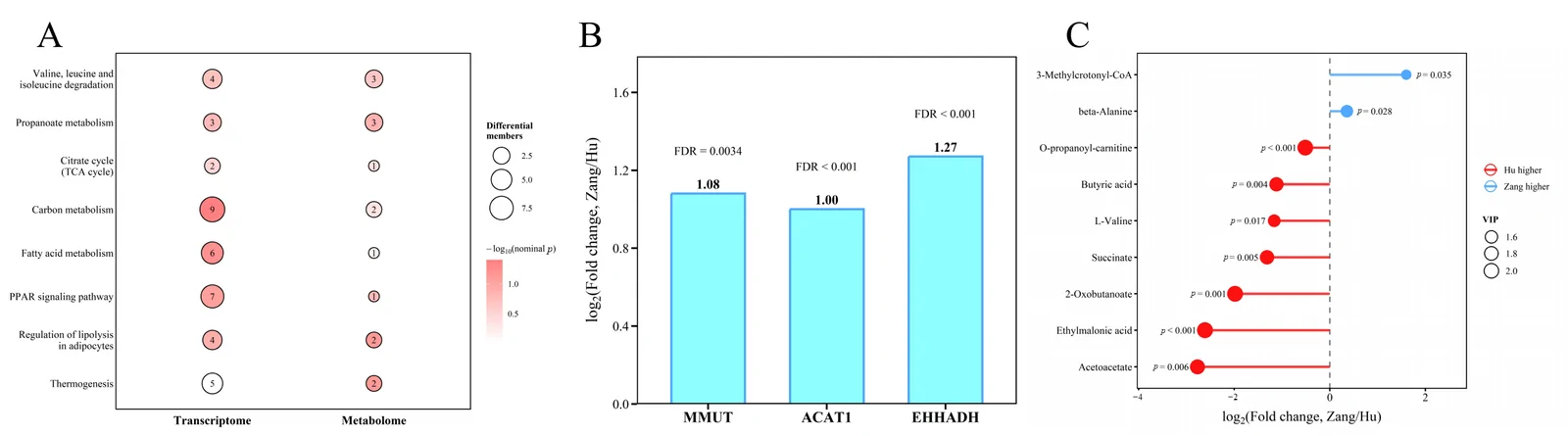
Integrated transcriptomic and metabolomic analysis identifies BCFA-associated metabolic pathways in Hu and Zang sheep. (**A**) Integrated analysis of differential genes and metabolites associated with BCFA-related metabolic pathways. (**B**) Expression of representative genes involved in the selected metabolic pathways. (**C**) Effect-size plot of representative differential metabolites. The vertical dashed line in (C) indicates log_2_FC = 0 (FC = 1), representing no fold change between the Zang and Hu groups.

Integrated multi-omics analysis of Hu and Zang sheep showed that the differential genes and metabolites were mainly enriched in pathways related to branched-chain amino acid degradation, propionate metabolism, the TCA cycle, and lipid metabolism (Figure 5A). Among the candidate genes in these core pathways, *MMUT*, *ACAT1*, and *EHHADH* showed higher expression levels in Zang sheep than in Hu sheep (Figure 5B). Analysis of representative differential metabolites showed that 2-oxobutanoate, succinate, acetoacetate, O-propanoyl-carnitine, and L-valine were more abundant in the Hu group, whereas 3-methylcrotonyl-CoA and beta-alanine were more abundant in the Zang group (Figure 5C).

To assess whether this metabolic module was specific to the Hu/Zang comparison, representative metabolic nodes from three independent comparison systems were further integrated, including ALT/TLF, Hu/Zang, and Tan CF/PF (Supplementary Figure S3). The results showed that metabolites related to BCAA degradation, propionate metabolism, and the TCA cycle exhibited coordinated changes across different genetic backgrounds and nutritional conditions.

### 3.6. Metabolomic Changes in the Longissimus Dorsi Muscle of Tan Sheep Under Different Feeding Conditions and Cross-Dataset Comparison

**Figure 6:**
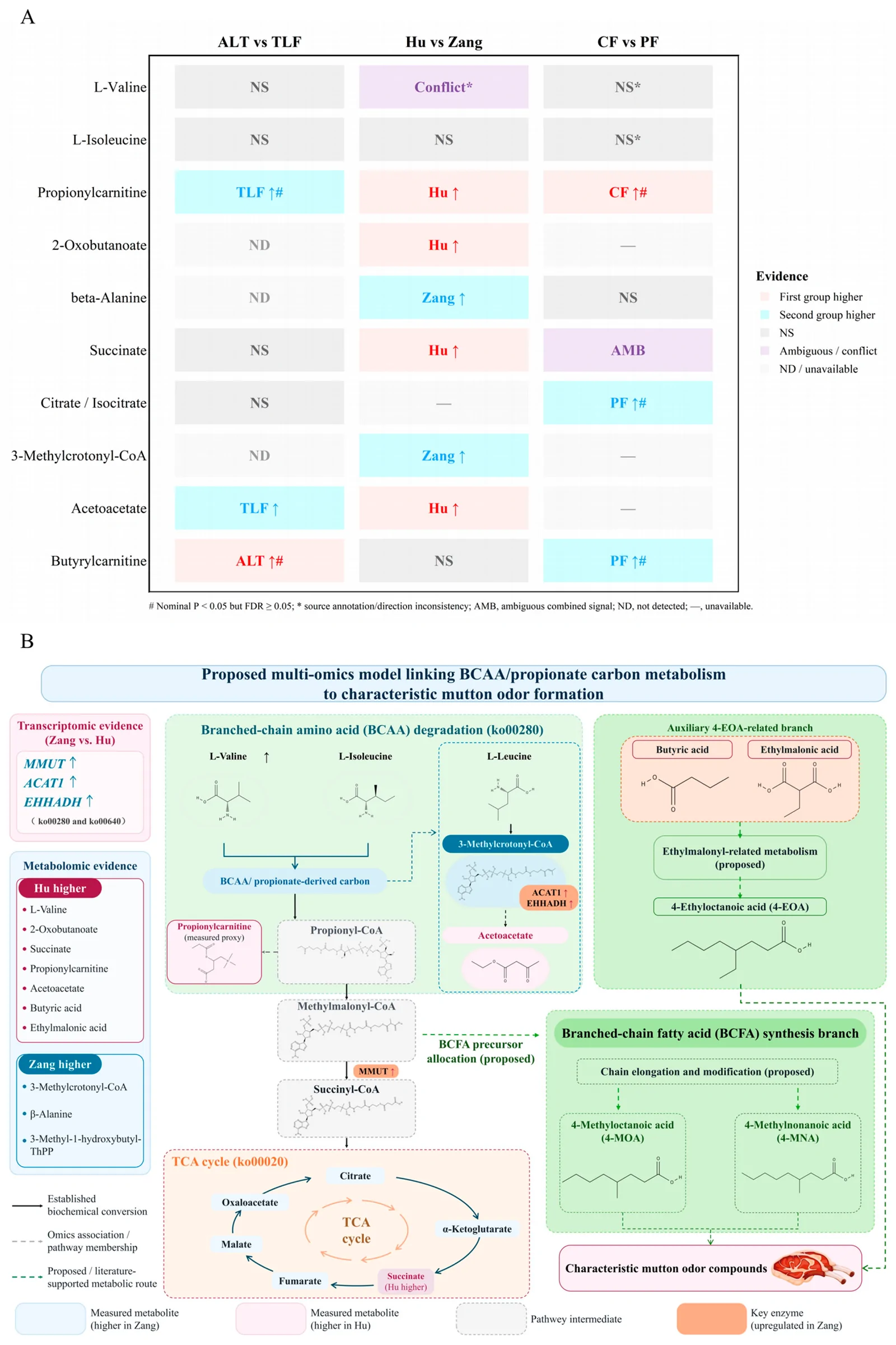
Cross-dataset evidence supporting the BCAA–short-chain carbon–TCA metabolic module and proposed BCFA-associated metabolic model. (**A**) Cross-dataset evidence matrix summarizing representative metabolic nodes in ALT vs. TLF, Hu vs. Zang, and CF vs. PF comparisons. (**B**) Proposed metabolic model integrating BCAA degradation, propanoate metabolism, the TCA cycle, and pathways related to 4-MOA, 4-MNA, and 4-EOA formation. Solid black lines indicate established biochemical reactions, grey dashed lines indicate omics-supported associations, and green dashed lines indicate proposed metabolic connections. Red and blue indicate higher abundance in the corresponding comparison groups as indicated. #, nominal *p* < 0.05 but FDR ≥ 0.05; *, source annotation or direction inconsistency; AMB, ambiguous combined signal; ND, not detected; —, unavailable. The ↑ symbol indicates higher metabolite abundance or gene expression in the indicated group relative to the other group in the corresponding comparison. Arrows connecting pathway nodes in (B) indicate biochemical reactions or metabolic relationships, as specified by the line styles.

Based on the metabolic evidence obtained from the different comparison systems, key metabolic nodes related to BCFA formation were further selected. A cross-dataset BCAA–propionate–TCA metabolic module was then constructed (Figure 6A). Entries marked with # in Figure 6A had nominal *p* < 0.05 but FDR ≥ 0.05 and were retained as exploratory observations.

Based on these results, we further constructed a candidate metabolic association model that included BCAA degradation, propionate metabolism, the TCA cycle, and metabolic branches related to 4-MOA, 4-MNA, and 4-EOA. Known biochemical relationships were incorporated to illustrate the potential links between these metabolic processes and the formation of the three BCFAs (Figure 6B). The model included candidate genes such as *MMUT*, *ACAT1*, and *EHHADH*. Potential metabolic connection nodes, including propionyl-CoA, methylmalonyl-CoA, and succinyl-CoA, were also indicated based on established biochemical reactions.

## 4. Discussion

This study used differences in the accumulation of 4-MOA, 4-EOA, and 4-MNA among sheep populations as the phenotypic basis and integrated muscle metabolomic and transcriptomic data from different ecological origins, feeding conditions, and genetic backgrounds. The aim was to identify metabolic processes and candidate regulatory factors associated with the formation and accumulation of these three odor-related BCFAs.

Although the datasets were derived from different biological contexts, the major metabolic changes consistently involved BCAA-related metabolism, short-chain carbon metabolism, the TCA cycle, and lipid metabolism. This finding suggests that BCFA formation may be closely related to the utilization and partitioning of branched-chain and short-chain carbon sources between central energy metabolism and lipid metabolism.

Among the candidate genes, *MMUT*, *ACAT1*, and *EHHADH* are involved in methylmalonyl-CoA utilization, acetyl-CoA/acetoacetyl-CoA metabolism, and fatty acid oxidation, respectively. These genes therefore provide candidate molecular links between differences in carbon source utilization and lipid metabolism and the formation of odor-related BCFAs under different biological and production conditions.

### 4.1. Accumulation Characteristics of Odor-Related BCFAs in Different Sheep Populations

As consumer expectations for the sensory and eating quality of meat continue to increase, flavor has become an important factor in meat quality evaluation and consumer choice. Sheep meat has a distinct species-specific flavor, but an excessively strong muttony odor can reduce its acceptance among some consumers. Therefore, reducing undesirable odor while retaining the characteristic flavor of sheep meat has become an important issue in meat quality improvement and flavor regulation [22]. Previous studies have shown clear population-specific differences in meat quality and flavor composition among sheep breeds. Li et al. [23] reported that different types of Tibetan sheep showed distinct differences in fatty acid composition, amino acid profiles, and volatile flavor compounds. Their muscle energy utilization and lipid and amino acid metabolism also differed among populations. Therefore, direct quantification of 4-MOA, 4-EOA, and 4-MNA can provide useful information on the composition and accumulation of odor-related BCFAs in different sheep populations.

In this study, 4-MOA, 4-EOA, and 4-MNA were quantified in the LD muscle of five sheep populations: ALT, TLF, NM, Tan, and Hu. All three BCFAs were below the limits of detection in ALT, TLF, and NM sheep. In Tan sheep, 4-MOA and 4-EOA were detected, whereas 4-MNA was below the detection limit. All three BCFAs were detected in Hu sheep. The concentrations of 4-MOA and 4-EOA were also markedly higher in Hu sheep than in Tan sheep. Zhang et al. [24] investigated four Chinese indigenous sheep breeds and found significant breed differences in 4-MOA, 4-EOA, and 4-MNA, with different distribution patterns among breeds. Compared with their findings, the present study showed a different pattern in the detection and abundance of these three BCFAs. Previous studies have also indicated that, in addition to breed, factors such as age and nutritional status can influence the accumulation of 4-alkyl BCFAs [9].

These findings suggest that the formation and deposition of 4-MOA, 4-EOA, and 4-MNA may be influenced by genetic background, ecological environment, and nutritional conditions. Differences in physiological and metabolic status under different biological and production conditions may also contribute to variation in BCFA phenotypes. All three target BCFAs were below the limits of detection in ALT, TLF, and NM sheep. The muscle metabolomic profiles of ALT and TLF sheep were further compared to examine metabolic variation between populations with contrasting ecological adaptation backgrounds and the same BCFA detection pattern.

### 4.2. BCFA-Related Metabolic Characteristics of Sheep from Different Ecological Origins

Sheep living in different ecological environments face different physiological demands for thermoregulation, energy utilization, and maintenance of metabolic homeostasis. These differences may be accompanied by changes in muscle metabolism and meat quality traits [25]. Liu et al. [26] compared Tibetan sheep raised at altitudes of 2900, 4000, and 5100 m and found clear differences in meat quality and muscle metabolic profiles among the three altitude groups. Carnitine derivatives increased with altitude, suggesting that long-term ecological differences are closely associated with muscle energy and lipid metabolism. Yang et al. [27] performed an integrated phenotypic, proteomic, and metabolomic analysis of longissimus dorsi muscle from ALT and Turpan Black sheep. They reported differences in meat quality traits and muscle molecular characteristics between the two breeds, with differential proteins and metabolites mainly involving lipid metabolism, amino acid metabolism, and stress-response networks. Tian et al. [28] conducted a multi-tissue metabolomic study of cold-tolerant ALT and heat-tolerant Turpan sheep. Under cold conditions, energy metabolism was redistributed across tissues, accompanied by changes in glycolysis and thermogenesis-related metabolism in adipose tissue, as well as changes in the TCA cycle and unsaturated fatty acid biosynthesis. Studies under high-temperature conditions have also reported marked metabolic and redox responses. Wang et al. [29] compared physiological indices, serum antioxidant indicators, and serum metabolomic profiles among Tibetan, Hu, and Huanghuai sheep under non-heat-stress and heat-stress conditions. Under heat stress, Tibetan sheep had lower serum CAT, T-AOC, and T-SOD activities than Hu sheep. Antioxidant-related metabolites, including creatine, ornithine, L-arginine, and guanosine, were decreased, whereas 3-nitrotyrosine was increased. Together, these studies indicate that ecological factors such as temperature and altitude are associated with adjustments in energy utilization, lipid metabolism, and redox status across muscle, multiple tissues, and serum.

In the present study, both ALT and TLF sheep showed low levels of odor-related BCFA accumulation, but their LD metabolic profiles differed markedly. L-octanoylcarnitine, carnitine C4:0, and isobutyric acid were more abundant in ALT sheep, whereas acetoacetic acid was more abundant in TLF sheep. The differential metabolites were associated with BCAA-related metabolism, fatty acid metabolism, and propionate-related metabolic processes. These findings suggest that similar BCFA accumulation phenotypes may arise from different patterns of energy utilization and lipid metabolism under different ecological backgrounds. Acylcarnitines are involved in the transport and oxidation of fatty acids and other acyl substrates, and changes in their composition and abundance are closely associated with mitochondrial fatty acid utilization and cellular energy homeostasis [30]. Acetoacetic acid is one of the major ketone bodies and can serve as an alternative energy substrate for oxidative metabolism. Its abundance is therefore closely related to fatty acid catabolism and energy substrate utilization [31]. Isobutyric acid is more directly linked to BCAA metabolism. In ruminants, dietary valine supplementation has been associated with increased isobutyric acid in rumen fluid, while the carbon skeleton derived from valine degradation can enter downstream metabolism through isobutyryl-CoA [32,33]. Thus, the changes observed in representative metabolites of ALT and TLF sheep do not appear to reflect a single metabolic process. Instead, they suggest coordinated differences in fatty acid utilization, ketone body metabolism, and BCAA-related short-chain carbon metabolism, indicating distinct patterns of energy substrate utilization and metabolic partitioning between sheep from different ecological origins.

The changes in L-octanoylcarnitine and carnitine C4:0 observed in ALT sheep are related to fatty acid oxidation and mitochondrial acyl transport, whereas the change in acetoacetic acid in TLF sheep is associated with ketone body utilization and energy substrate switching. These processes are all closely linked to mitochondrial energy metabolism [30,31]. In addition to energy production, mitochondrial fatty acid oxidation is connected with NADPH supply, antioxidant defense, and redox homeostasis, creating a close metabolic link between energy metabolism and cellular stress responses [34]. Tian et al. [28] found that cold exposure in ALT sheep was accompanied by enhanced thermogenesis and mitochondrial ROS production in brown adipose tissue, together with changes in metabolites related to endogenous antioxidant capacity in the digestive tract. Wang et al. [29] also reported reduced serum antioxidant enzyme activities and several antioxidant-related metabolites in heat-stressed Tibetan sheep, together with an increase in the oxidative stress marker 3-nitrotyrosine. Although ALT and TLF shared the same BCFA detection pattern, their muscle profiles differed in metabolites related to lipid, ketone body, and BCAA metabolism. These differences may affect precursor availability. Dietary composition and feeding conditions may further influence substrate supply and utilization.

### 4.3. BCFA-Related Metabolic Characteristics of Sheep Under Different Feeding Conditions

Feeding systems and dietary composition are important external factors that influence nutrient substrate utilization, the rumen fermentation environment, and tissue metabolic status in ruminants [35]. Different nutritional levels can also alter the composition of muscle fatty acids, amino acids, and flavor precursors. Sun et al. [36] compared supplemented and restricted feeding in Hu sheep and found that supplementation increased the concentrations of several volatile flavor compounds and amino acids in muscle, whereas restricted feeding was associated with enhanced lipolysis and reduced intramuscular fat. Xie et al. [37] reported that different feed intake levels in Gangba sheep altered carbohydrate, lipid, and energy metabolism. Moderate feed restriction followed by compensatory feeding promoted the accumulation of flavor precursors such as free amino acids and monounsaturated fatty acids. These findings indicate that the effects of nutritional conditions on sheep meat flavor are closely associated with changes in muscle lipid, amino acid, and energy metabolism.

In the present study, 4-MOA and 4-EOA were detected in Tan sheep muscle, whereas 4-MNA was below the detection limit, showing a BCFA profile distinct from those of ALT/TLF and Hu sheep. Metabolomic analysis showed that propionylcarnitine was more abundant in the CF group, whereas citric acid, isocitric acid, and butyrylcarnitine were more abundant in the PF group. L-valine, L-isoleucine, L-leucine, and succinic acid/methylmalonic acid also showed varying degrees of change between the two groups, suggesting that nutritional conditions can influence carbon utilization and energy metabolic networks related to BCFA formation. The composite scores for BCAA degradation and propionate-related metabolism did not differ significantly between the CF and PF groups, whereas the TCA cycle showed a significant difference. Propionylcarnitine is closely associated with the formation and transport of intracellular propionyl-CoA. Propionyl-CoA can also be generated from the degradation of BCAAs such as valine and isoleucine and is connected to the TCA cycle through methylmalonyl-CoA and succinyl-CoA [33]. Citric acid, isocitric acid, and succinic acid are key intermediates of the TCA cycle. Therefore, the changes in these metabolites and in the TCA cycle composite score are more likely to reflect differences in energy substrate utilization and central carbon metabolism under different feeding systems, rather than an isolated overall change in BCAA degradation or propionate-related metabolism.

Cheng et al. [38] found higher ruminal isobutyrate concentrations and greater 4-MNA deposition in concentrate-fed Bashbay sheep than in grazing sheep, accompanied by differences in rumen microbiota and lipid metabolism. In the public Tan dataset, CF and PF sheep also differed in propionylcarnitine, TCA cycle intermediates, and butyrylcarnitine. Changes in substrate supply and utilization may therefore contribute to variation in BCFA accumulation under different feeding systems.

Metabolic changes induced by nutritional conditions may also be accompanied by adjustments in muscle redox status. Meng et al. [35] found that changes in the rumen environment induced by a high-concentrate diet occurred together with oxidative stress and disturbed lipid metabolism in Hu sheep muscle, whereas supplementation with disodium fumarate improved both antioxidant status and lipid metabolism. Xie et al. [37] also observed different redox and energy metabolic states under different feed intake levels during compensatory feeding, and these changes were associated with differences in flavor precursor accumulation. In addition, Li et al. [39,40] showed that dietary supplementation with prickly ash seed improved antioxidant capacity in the serum and muscle of Hu sheep and altered fatty acid, amino acid, and muscle metabolite profiles. Their subsequent study further showed that this nutritional intervention affected the expression of genes related to muscle fatty acid metabolism and the composition of volatile flavor compounds. Therefore, nutrition-induced changes in redox regulation may contribute to alterations in lipid and energy metabolism and thereby influence the formation and retention of flavor precursors. For odor-related BCFAs, the Tan sheep results in the present study point directly to changes in substrate supply, short-chain carbon metabolism, and central energy metabolism. The utilization and metabolic partitioning of these nutrient-derived carbon skeletons may also differ among genetic backgrounds, suggesting that genetic background may contribute to population differences in BCFA accumulation.

### 4.4. BCFA-Related Metabolic Characteristics and Candidate Genes in Sheep with Different Genetic Backgrounds

Sheep with different genetic backgrounds can show clear differences in meat quality and muscle metabolism even under similar feeding conditions. Xu et al. [41] compared Oula sheep and Panou Tibetan sheep under the same grazing conditions and found differences in intramuscular fat content, fatty acid composition, and meat quality traits. Integrated lipidomic and transcriptomic analyses identified glycerophospholipid metabolism and several lipid metabolism-related genes, including *SCD5*, *PLA2G4E*, *PCK1*, and *ELOVL2*, as important features distinguishing the two breeds. Similarly, Ma et al. [42] compared Gansu Alpine fine wool sheep and Tianhua mutton sheep in the Qilian Mountains and reported clear differences in tenderness, amino acid composition, and fatty acid profiles. Their integrated transcriptomic and metabolomic analyses identified *FABP4*, *PLIN1*, *PCK1*, and L-valine as molecular features associated with differences in muscle metabolism and meat quality. These studies indicate that genetic differences among sheep breeds are accompanied by changes in muscle lipid deposition, amino acid utilization, and energy metabolism. These processes also provide an important metabolic background for the formation and utilization of flavor precursors in different sheep populations.

In the present study, Hu sheep showed a relatively high accumulation of odor-related BCFAs. All three BCFAs, 4-MOA, 4-EOA, and 4-MNA, were detected, and the concentrations of 4-MOA and 4-EOA were markedly higher than those in Tan sheep. Independent multi-omics analysis of Hu and Zang sheep showed higher abundances of O-propanoyl-carnitine, ethylmalonic acid, 2-oxobutanoate, butyric acid, succinate, acetoacetate, and L-valine in Hu sheep, whereas 3-methylcrotonyl-CoA and beta-alanine were more abundant in Zang sheep. These differences were mainly associated with BCAA degradation, propionate and butyrate metabolism, the TCA cycle, and fatty acid metabolism. The observed metabolic changes were therefore concentrated in interconnected processes involving branched-chain carbon utilization, short-chain carbon metabolism, and lipid metabolism. The metabolic differences observed in the public Hu/Zang cohort point to precursor utilization and lipid metabolism as possible contributors to variation in BCFA accumulation.

Analysis of the public Hu/Zang dataset identified *MMUT*, *ACAT1*, and *EHHADH* as candidate genes, with higher expression in Zang sheep. *MMUT* converts methylmalonyl-CoA to succinyl-CoA, linking its utilization to the TCA cycle. In sheep, cobalt–vitamin B12 deficiency reduced methylmalonyl-CoA mutase activity and altered tissue fatty acid composition [43]. *ACAT1* participates in acetyl-CoA/acetoacetyl-CoA metabolism, and single-cell analysis of the sheep rumen identified it among the ketogenesis-related genes expressed in rumen epithelial cells [44]. *EHHADH* participates in peroxisomal fatty acid β-oxidation. A feeding study in Tan sheep reported altered *EHHADH* expression alongside reduced intramuscular fat content [45]. These metabolic roles provide a rationale for investigating associations between the three genes and BCFA variation.

In the public Hu/Zang cohort, *MMUT*, *ACAT1*, and *EHHADH* were more highly expressed in Zang sheep, whereas O-propanoyl-carnitine, acetoacetate, succinate, and L-valine were more abundant in Hu sheep. This pattern may reflect differences in substrate supply and utilization. The known functions of the three genes make them plausible candidates for studying the metabolic basis of BCFA variation. The Hu sheep in this dataset were independent of those used for BCFA quantification in our study. Testing whether these expression differences are associated with the BCFA phenotypes reported here requires gene expression and BCFA measurements in the same animals. Functional studies would then help determine whether the genes affect BCFA formation or accumulation.

### 4.5. Cross-Dataset Integration Reveals Shared BCFA-Related Metabolic Networks and Candidate Regulatory Nodes

Across the ALT/TLF, Hu/Zang, and Tan CF/PF comparisons, metabolic differences involved BCAA metabolism, short-chain carbon metabolism, the TCA cycle, and lipid utilization. L-valine, propionylcarnitine, acetoacetate, and succinate were among the metabolites highlighted, although the specific compounds and their directions of change varied among datasets. The Hu/Zang transcriptomic analysis also identified *MMUT*, *ACAT1*, and *EHHADH* as candidates within these pathways. These observations suggest that substrate utilization may contribute to BCFA variation. However, the studies used independent cohorts with different animal backgrounds and experimental conditions. Animal age, feeding conditions, and laboratory procedures may also influence the observed patterns, limiting the attribution of BCFA differences to individual environmental, nutritional, or genetic factors.

BCAA degradation provides an important carbon source connection within this metabolic network. The carbon skeletons generated from valine and isoleucine degradation can enter propionyl-CoA-related metabolism, be converted through methylmalonyl-CoA to succinyl-CoA, and then enter the TCA cycle. Leucine degradation proceeds through intermediates such as 3-methylcrotonyl-CoA and ultimately produces acetyl-CoA and acetoacetate [46,47]. Therefore, the changes in L-valine, 3-methylcrotonyl-CoA, propionylcarnitine, acetoacetate, and succinate observed across the datasets are biochemically connected. Together, they reflect the utilization of BCAA-derived carbon skeletons between short-chain carbon metabolism and central energy metabolism. In ruminants, BCAAs are involved not only in protein synthesis, but their degradation products can also enter glucose and lipid metabolic networks. This creates a close connection among amino acid supply, energy utilization, and lipid synthesis [46].

This carbon partitioning is also directly related to the formation of 4-alkyl BCFAs. Previous studies suggest that the formation of 4-MOA and 4-MNA is closely associated with propionyl-CoA and methylmalonyl-CoA derived from propionate. Methylmalonyl-CoA may provide branched carbon units for subsequent fatty acid chain elongation [48]. One study showed that modulation of rumen microbial and hepatic metabolism reduced ruminal propionate levels and was accompanied by significant decreases in muscle 4-MOA, 4-EOA, and 4-MNA. Changes in the hepatic metabolome mainly involved amino acid metabolism, carnitine-related metabolism, and lipid catabolism, suggesting that BCFA precursors may have been redirected from lipid synthesis toward other metabolic pathways [48]. In contrast, the biosynthetic pathway of 4-EOA has not yet been fully clarified. Current evidence suggests that butyryl-CoA can be carboxylated to ethylmalonyl-CoA, which may provide an ethyl-branched carbon source for 4-EOA synthesis [22]. Thus, although 4-MOA, 4-MNA, and 4-EOA are all odor-related 4-alkyl BCFAs, they may differ in their dependence on specific precursor carbon sources. This may partly explain why the three BCFAs did not always show the same accumulation pattern across sheep populations.

Metabolite differences in the ALT/TLF and Tan CF/PF datasets also suggest variation in substrate supply and utilization. Controlled comparisons could help distinguish how environmental adaptation, diet, and genetic background influence these processes.

All three BCFAs were below the limits of detection in ALT, TLF, and NM sheep, whereas 4-MOA and 4-EOA were detected in Tan sheep. In Hu sheep, all three BCFAs were detected, and 4-MOA and 4-EOA showed relatively high levels of accumulation. All three target BCFAs were below the limits of detection in NM sheep, showing the same detection pattern as ALT and TLF and differing from Tan and Hu. This finding provides phenotypic information from another indigenous population and shows that the pattern of non-detection observed in this study was not restricted to ALT and TLF. Notably, ALT and TLF differed in muscle acylcarnitines, acetoacetate, and isobutyric acid despite sharing the same BCFA detection pattern, indicating that similar detection patterns can coexist with different muscle metabolic profiles. The NM results therefore provide an additional phenotypic reference for population comparisons, while the candidate metabolic processes identified across datasets offer directions for further investigation of the metabolic basis of this phenotype in NM sheep. The environmental, nutritional, and genetic multi-omics results suggest that these phenotypic differences are unlikely to result from changes in a single metabolic pathway. Instead, they may be associated with differences in the partitioning of BCAA-derived, short-chain, and lipid-derived carbon between energy metabolism and fatty acid formation. Based on the metabolic nodes repeatedly identified across datasets, this study proposes BCAA-related metabolism, short-chain carbon metabolism, and the TCA cycle as important metabolic modules linking multiple influencing factors with BCFA accumulation. The Hu/Zang transcriptomic analysis identified *MMUT*, *ACAT1*, and *EHHADH* as candidate genes for further investigation within these pathways.

Linking the BCFA profiles reported here with sensory scores could support product categories with mild or pronounced mutton flavor. Consumer testing in target markets would help guide branding and product selection for retail and restaurant use [49], while willingness-to-pay surveys could assess commercial value. Electronic noses have been used to distinguish sheep and goat meat from their volatile fingerprints [50]. For rapid flavor screening, sensor models could be calibrated against GC–MS measurements and sensory scores. Their classification accuracy, sampling time, and performance across production batches could then be evaluated under slaughter-line conditions to assess their suitability for carcass sorting.

### 4.6. Limitations

The datasets differed in animal background and analytical procedures, so the cross-study findings were interpreted as hypotheses about related metabolic pathways. MMUT, ACAT1, and EHHADH were identified from the public Hu/Zang data and remain to be validated in the five populations studied here. All three target BCFAs were below the LOD in the ALT/TLF cohort, while the public datasets involved animals independent of our BCFA cohorts; thus, paired metabolite–BCFA correlations could not be estimated. Matched molecular and BCFA measurements, together with functional and flux studies, would help test the proposed mechanisms, including carbon allocation.

## 5. Conclusions

BCFA measurements showed distinct detection patterns among the five sheep populations. Analysis of the ALT/TLF muscle metabolome and the public Hu/Zang and Tan CF/PF datasets identified differences involving BCAA degradation, short-chain carbon metabolism, the TCA cycle, and lipid metabolism. Differences in precursor supply and utilization may help explain the BCFA patterns observed here. Controlled studies are needed to determine how environment, diet, and genetic background influence these processes and BCFA accumulation. The enzymes encoded by *MMUT*, *ACAT1*, and *EHHADH* are involved in the conversion of methylmalonyl-CoA to succinyl-CoA, acetyl-CoA/acetoacetyl-CoA metabolism, and fatty acid β-oxidation, respectively. Reanalysis of the public Hu/Zang transcriptomic data identified *MMUT*, *ACAT1*, and *EHHADH* as candidate genes related to these metabolic processes. The gene-expression profiles and the BCFA measurements reported here were obtained from separate animal cohorts. Together with the BCFA measurements, these results provide a basis for further research on BCFA-related flavor variation and its nutritional and genetic regulation in sheep.

## Figures and Tables

**Table 1:** Breed identities, sampling information, and feeding conditions of the five sheep populations used for BCFA quantification.

Population	Breed Identity and Animal Source	Rearing and Sampling Locations	Sampling Season and Environmental Conditions	Feeding System and Main Dietary Components
ALT	Altay sheep; Xinjiang Uygur Autonomous Region	Fuhai County, Altay Prefecture, Xinjiang Uygur Autonomous Region, China	Winter	Grazing and supplementary feeding; pasture, corn
TLF	Turpan sheep; Xinjiang Uygur Autonomous Region	Turpan City, Xinjiang Uygur Autonomous Region, China	Winter	Grazing and supplementary feeding; pasture, corn
NM	Inner Mongolian sheep; Inner Mongolia Autonomous Region	Sunite Left Banner, Xilingol League, Inner Mongolia Autonomous Region, China	Winter	Grazing and supplementary feeding; pasture, corn
Tan	Tan sheep; Ningxia Hui Autonomous Region	Yinchuan, Ningxia Hui Autonomous Region, China	Winter	Grazing and supplementary feeding; pasture, corn
Hu	Hu sheep; Zhejiang Province	Huzhou City, Zhejiang Province, China	Winter	Housing; corn stalk, corn

## Data Availability

The original data presented in this study are openly available in Figshare at https://figshare.com/s/b50b625175825df37863 (accessed on 1 November 2026). For peer review, the data are available through a private Figshare link. The Hu/Zang differential-expression results and gene annotations used in this study were obtained from Wang et al. [18] and the associated Supplementary Materials. The original RNA-seq reads are archived in the NCBI Sequence Read Archive under BioProject PRJNA1081808. The Hu/Zang metabolomic data and the Tan sheep metabolomic data were obtained from the previously published studies of Wang et al. [18] and Yang et al. [19], respectively, and are available through the original publications and their associated Supplementary Materials. All other data supporting the findings of this study are included in the article and its Supplementary Materials.

## Supplementary Materials

The following supporting information can be downloaded at https://www.mdpi.com/article/10.3390/foods15213964/s1. Figure S1: KEGG pathway annotation of differential metabolites between ALT and TLF sheep; Figure S2: KEGG pathway annotation of differential metabolites between Hu and Zang sheep; Figure S3: Cross-dataset comparison of representative metabolic nodes associated with BCAA degradation, propionate metabolism, and the TCA cycle in the ALT/TLF, Hu/Zang, and Tan CF/PF comparison systems.
