# Supplementary material for: Multi-Omics Insights into the Metabolic Basis of Variation in 4-Alkyl Branched-Chain Fatty Acids in Meat from Chinese Sheep Breeds
Source: Foods. 2026 Nov 4;15(21):3964. doi: 10.3390/foods15213964 (PMC13763203; doi:10.3390/foods15213964)
Supplement: Supplementary file 1 [file foods-15-03964-s001.zip › foods-4617300-supplementary.pdf]

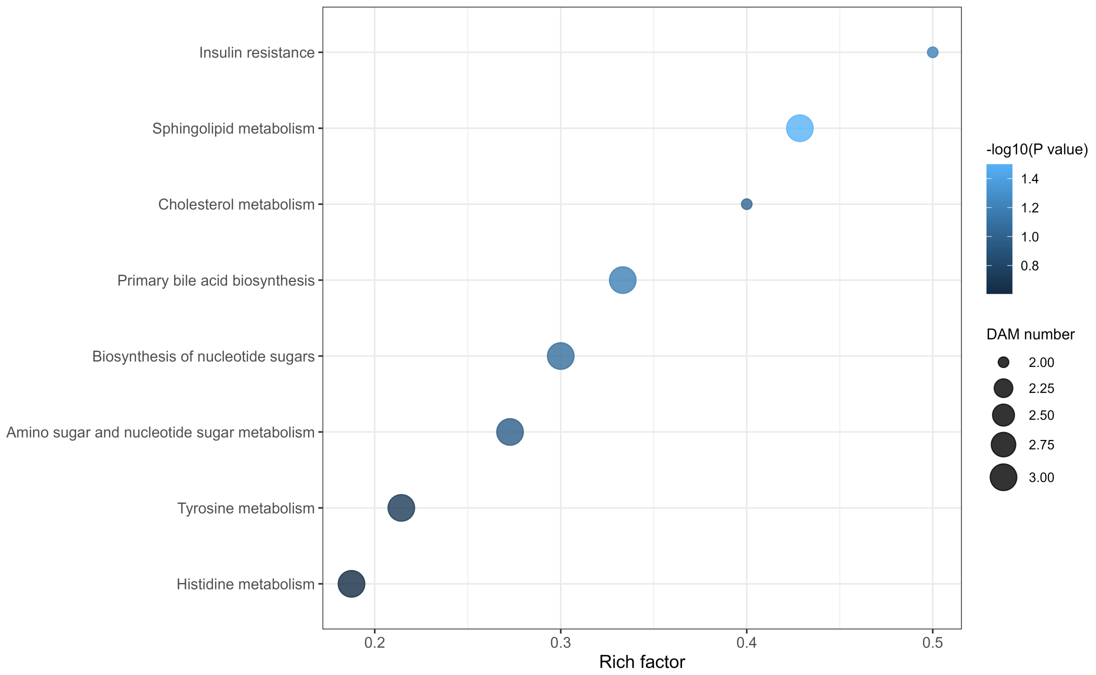

Supplementary Figure S1. KEGG pathway annotation of differential metabolites between ALT and TLF sheep.
Bubble plot showing KEGG pathways enriched by differential metabolites between ALT and TLF sheep. Dot size represents the number of differential metabolites assigned to each pathway, and color indicates the mean VIP value.

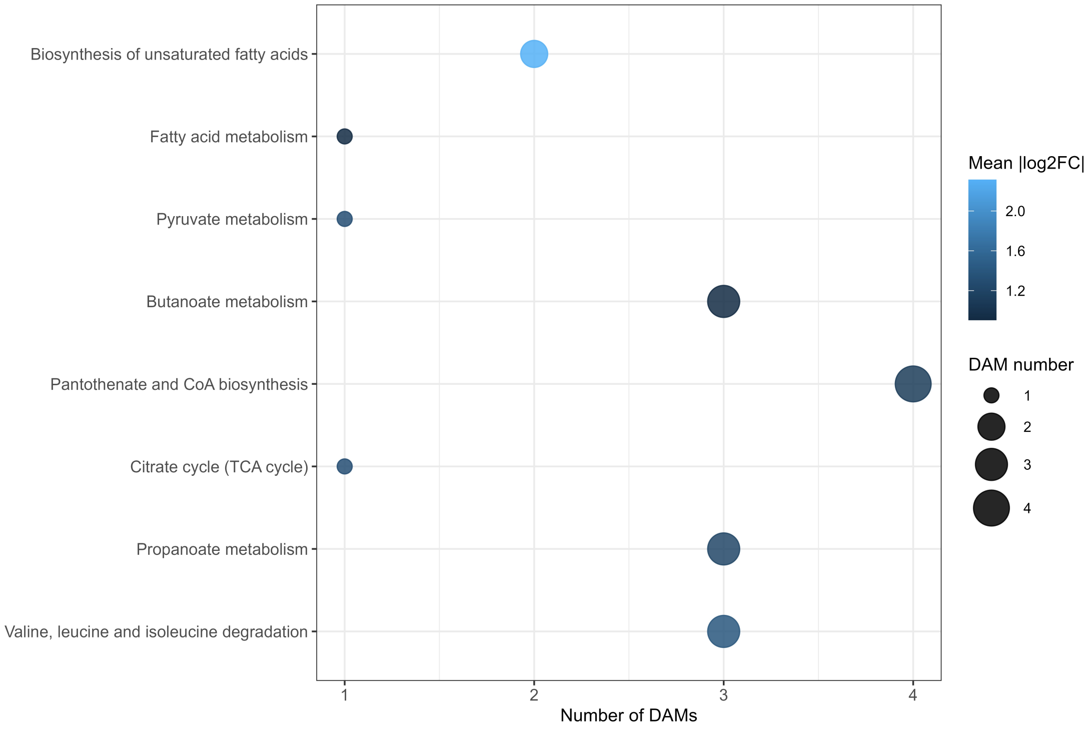

Supplementary Figure S2. KEGG pathway annotation of differential metabolites between Hu and Zang sheep.
Bubble plot showing KEGG pathways associated with differential metabolites between

Hu and Zang sheep. Dot size indicates the number of differential metabolites, and color indicates pathway-associated metabolite abundance changes.

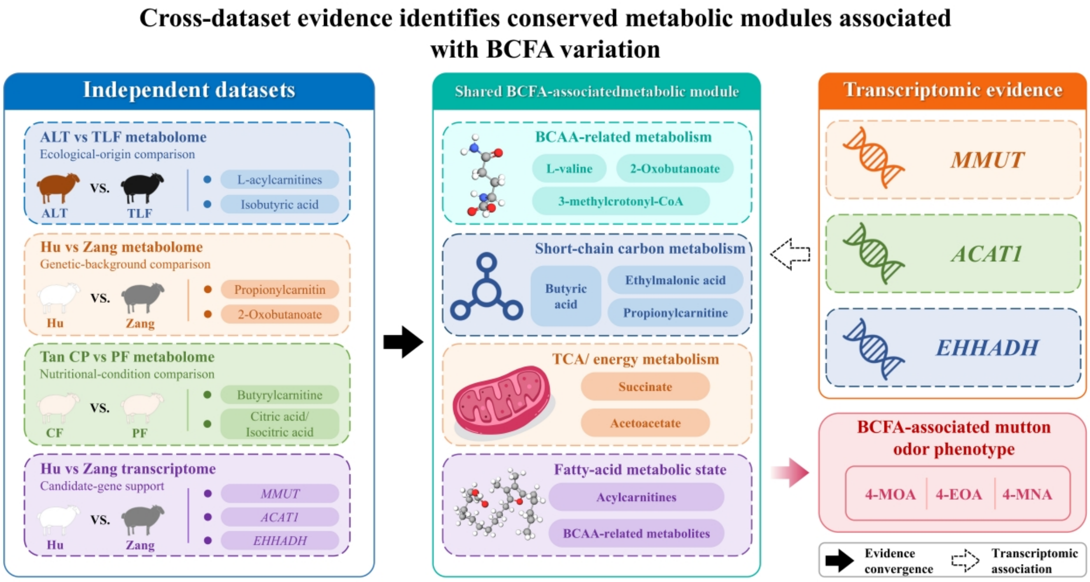

Supplementary Figure S3. Cross-dataset integration of BCFA-associated metabolic signatures across sheep populations, feeding conditions, and transcriptomic evidence. Comparison of representative metabolites involved in BCAA degradation, short-chain carbon metabolism, and TCA cycle among ALT vs TLF, Hu vs Zang, and CF vs PF Tan sheep datasets.
